# Deep sequencing-based transcriptome analysis of *Plutella xylostella *larvae parasitized by *Diadegma semiclausum*

**DOI:** 10.1186/1471-2164-12-446

**Published:** 2011-09-09

**Authors:** Kayvan Etebari, Robin W Palfreyman, David Schlipalius, Lars K Nielsen, Richard V Glatz, Sassan Asgari

**Affiliations:** 1 School of Biological Sciences, The University of Queensland, St Lucia QLD 4072 Australia; 2 Australian Institute for Bioengineering and Nanotechnology, The University of Queensland, St Lucia QLD 4072 Australia; 3 AgriScience Queensland, Department of Employment Economic Development and Innovation, Ecosciences Precinct, GPO Box 46, Brisbane 4001, Australia; 4 South Australian Research and Development Institute (SARDI), Entomology, Waite Road, Urrbrae, South Australia, 5064, Australia

## Abstract

**Background:**

Parasitoid insects manipulate their hosts' physiology by injecting various factors into their host upon parasitization. Transcriptomic approaches provide a powerful approach to study insect host-parasitoid interactions at the molecular level. In order to investigate the effects of parasitization by an ichneumonid wasp (*Diadegma semiclausum*) on the host (*Plutella xylostella*), the larval transcriptome profile was analyzed using a short-read deep sequencing method (Illumina). Symbiotic polydnaviruses (PDVs) associated with ichneumonid parasitoids, known as ichnoviruses, play significant roles in host immune suppression and developmental regulation. In the current study, *D. semiclausum *ichnovirus (*Ds*IV) genes expressed in *P. xylostella *were identified and their sequences compared with other reported PDVs. Five of these genes encode proteins of unknown identity, that have not previously been reported.

**Results:**

*De novo *assembly of cDNA sequence data generated 172,660 contigs between 100 and 10000 bp in length; with 35% of > 200 bp in length. Parasitization had significant impacts on expression levels of 928 identified insect host transcripts. Gene ontology data illustrated that the majority of the differentially expressed genes are involved in binding, catalytic activity, and metabolic and cellular processes. In addition, the results show that transcription levels of antimicrobial peptides, such as gloverin, cecropin E and lysozyme, were up-regulated after parasitism. Expression of ichnovirus genes were detected in parasitized larvae with 19 unique sequences identified from five PDV gene families including vankyrin, viral innexin, repeat elements, a cysteine-rich motif, and polar residue rich protein. Vankyrin 1 and repeat element 1 genes showed the highest transcription levels among the *Ds*IV genes.

**Conclusion:**

This study provides detailed information on differential expression of *P. xylostella *larval genes following parasitization, *Ds*IV genes expressed in the host and also improves our current understanding of this host-parasitoid interaction.

## Background

Endoparasitoids of the insect order Hymenoptera inject their eggs inside a host insect where they hatch and subsequently feed on the host until its death. For successful parasitism, endoparasitoids bring about a change in their hosts' conditions in favour of the developing parasitoid larvae. For this purpose, female wasps introduce secretions such as venom or ovary fluids, which may contain symbiotic viruses (polydnavirus (PDV) and/or virus-like particles), and other maternal factors, into the host [1]. In addition to suppression of the host immune system to protect the developing parasitoid, several studies have shown that parasitoids and their introduced maternal factors have significant effects on host metabolism and development such as plasma protein composition, food consumption, endocrine system activity and even on regulatory microRNA levels [2-8].

*Diadegma semiclausum *Hellén is an ichneumonid endoparasitoid that carries a PDV with a circular, double-stranded and segmented DNA genome encoding proteins that suppress the host immune response and cause developmental arrest/delay [9]. PDVs are the most highly characterized of the known mutualistic viruses [10], replicating only within the calyx cells of the reproductive tract of female wasps [11]. No virus replication occurs in parasitized larvae; however expression of encapsidated PDV genes induces different physiological modifications such as interruption of the larval endocrine system and suppression of the host immune system [12-15].

PDVs are classified into two genera based on their host wasp families; *Ichnovirus *containing ichnoviruses (IVs) and *Bracovirus *containing bracoviruses (BVs) [11,16]. These genera are morphologically distinct and their gene functions vary. Analysis of virion structural components from IVs indicates that the set of structural genes is conserved among wasps associated with IVs and might originate from an ancestral virus [17]. Recently, Bigot *et al*. suggested that IVs originated from ascoviruses by lateral transfer of ascoviral genes into wasp genomes [18]. Interestingly, the DNA that is encapsidated within PDVs appears more similar to that of eukaryotes than that of other viral genomes [19]. It has been demonstrated that less than 2% of encapsidated PDV genes have homologs in other viruses in contrast to the proviral or structural genes [20]. Most of the PDV DNA appear to be non-coding, except for some groups of genes that are involved in host immune suppression pathways [21]. IV genomes generally encompass more than 20 circular DNA segments ranging in size from 2-28 Kb with estimated total genome sizes ranging from 75 Kb to greater than 250 Kb [20].

Research so far has concentrated mainly on individual genes or small defined groups of host or PDV genes, to explore their function or differential expression following parasitization. Deep sequencing data can provide extensive information about host-parasitoid interactions at the transcriptome level. The large amount of sequencing data that can readily be produced by next-generation sequencing platforms, such as the Illumina GAII, reduces the need for prior sequence knowledge for gene expression profiling and are now making direct sequencing approaches the method of choice for whole transcriptome analysis in many species [22-26]. The large numbers of short reads produced by next-generation sequencers provide opportunities for development of new applications where sequencing only a portion of a molecule is sufficient. However, the analysis of transcriptome data produced by these technologies for organisms with limited genomic information still presents challenges because the sequenced fragments must be aligned against existing good quality reference genomes [27].

In this study, we used deep sequencing to explore the impact of *D. semiclausum *parasitization on its host, Diamondback moth *Plutella xylostella *L. (Lepidoptera, Plutellidae), a notorious pest of cruciferous plants. *P. xylostella *has developed resistance to many groups of chemical insecticides and also *Bacillus thuringiensis *endotoxin [28,29]. This has made *P. xylostella *one of the world's most destructive insect pests and the estimated global cost of controlling this insect is around US$1 billion annually [28]. This emphasizes the necessity for the continued development of innovative alternative control measures and resistance management strategies. Parasitoids or parasitoid-produced regulatory molecules can be used to improve conventional pest control strategies in sustainable agriculture. We have used a deep sequencing approach to give a comprehensive view of immune- and metabolic-related genes that are differentially expressed in parasitized versus non-parasitized *P. xylostella *larvae, revealing a significant number of *D. semiclausum *ichnovirus genes (*Ds*IV) which have not been reported previously. This type of study may facilitate new controls for pest larvae by identifying molecules that are crucial for larval immune defence, development, pesticide resistance and other important metabolic regulatory functions.

## Results and Discussion

### *P. xylostella* transcriptome profile

A transcriptome is the complete set of expressed RNA transcripts in one or more cells. Transcriptome profiling of organisms under stress or parasitization challenge helps us to obtain a better understanding of subsequent related cellular activities in organisms including growth, development, and immune defence. Recently, the newly developed deep sequencing approaches have significantly changed how the functional complexity of the transcriptome can be investigated [22,25,30].

**Figure 1:**
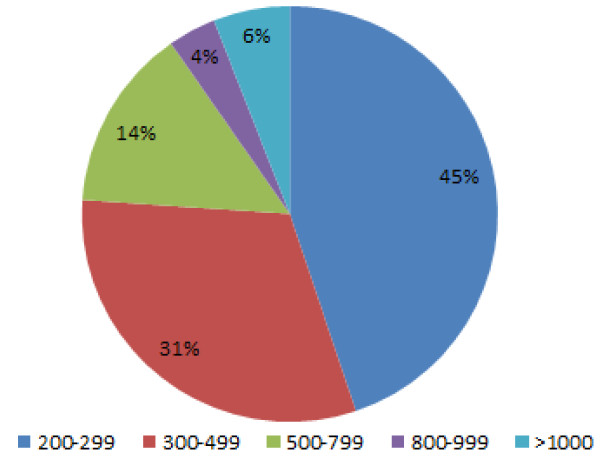
**Distribution of contig size in assembled *P. xylostella *transcriptome**. *De novo *assembly of RNAseq data by CLC genomic workbench generated 172,660 contigs between 100 and 10000 bp in length; with 35% of > 200 bp in length. Only contigs above 200 bp were selected for further analysis.

To analyse the transcriptome of *P. xylostella *host larvae following parasitization by *D. semiclausum*, RNA samples isolated from the whole host larvae at various time points after parasitization were pooled together prior to sequencing. This may lead to bias in the results (e.g. a gene may first be up-regulated and down-regulated subsequently); however, we aimed at obtaining an overview of what occurs during parasitism and generating a transcriptome of *P. xylostella *which has not been previously available and to isolate as many *Ds*IV genes as possible. Illumina GAII RNAseq deep sequencing analysis produced approximately 26.6 and 27.1 million single-end reads from RNA extracted from the whole body of non-parasitized (control) and parasitized larvae of *P. xylostella*, respectively. *De novo *assembly using the CLC Genomic Workbench produced 172,660 contigs with a minimum contig size of 100 bp; of these, 66% were between 100-199 bp and only 59,255 (34%) of the contigs were above 200 bp (Table 1). Only contigs above 200 bp were selected for further analysis and 6% of these had nucleotide lengths above 1000 bp (Figure 1). We found 4992 contigs that shared their greatest homology with *Tribolium castaneum *(Coleoptera) genes with a minimum E-value of 1e-06. *Bombyx mori *(silkworm) was another species with which a range of *P. xylostella *genes showed high levels of homology. One reason for the higher number of hits against the beetle genome is that three times more *T. castaneum *genes than *B. mori *genes are reported in databases.

**Table 1:** Summary of contig statistics resulting from Illumina deep sequencing of parasitized and non-parasitized *P. xylostella *larvae

Parameters	Number
Total *de novo *assembled contigs with CLC software	172,660
Contigs used for BLAST (cut-off: above 200 bp length)	59,255
Best BLAST matches (cut-off: E-value > E-6)	26119
No Hits (contigs without any BLAST match)	33136
Not mapped with any Gene Ontology (GO) database	6087
Annotated sequences (cut off: GO weight 5E value > E-6)	20704

**Figure 2:**
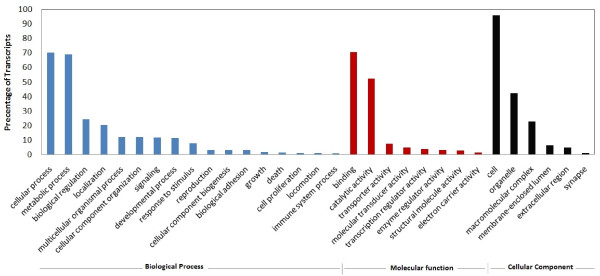
**GO annotation of consensus sequences (Level 2)**. 20704 sequences were annotated by B2GO software. This program categorized 14075 contigs in biological process, 18127 contigs in molecular function and 10048 contigs in cellular components. The data from InterPro terms, EC codes and KEGG were merged with GO terms for a wide functional range cover in B2GO annotation.

The short-read data generated by deep sequencing may be problematic when annotating alternative splice variants [24]. However, many gene expression profiling studies that use high-throughput sequencing can also provide valuable annotation information, such as existence of novel genes, exons or splice events which can be used to annotate putative gene sequences for other species. For instance, 39% of the reads from the brain transcriptome of the wasp, *Polistes metricus*, were matched to the honeybee (*Apis mellifera*) genome sequence and EST resources, for annotation [31]. Using a series of filtering and critical cut-off values for BLAST E-value and gene ontology (GO) weighting, 20,704 sequences were annotated by B2GO software http://www.blast2go.org through UniProt KB/TrEMBL and other available databases. GO-annotated consensus sequences were assigned to biological process clusters such as cellular component and molecular function, and distributed among various sub-categories such as metabolism, growth, development, apoptosis, immune defense, molecular processing, signal transduction, transcription regulator activity, catalytic activity etc. (Figure 2).

**Figure 3:**
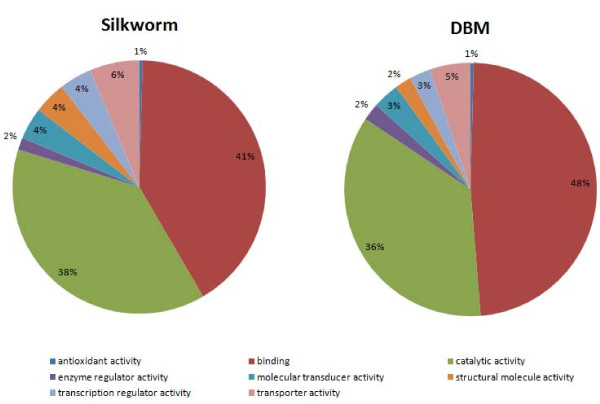
**Comparison of *P. xylostella *(DBM; diamondback moth) transcriptome pattern with that of *B. mori *(silkworm), based on 8 different molecular function Gene Ontology (GO) terms (Level 2)**. Silkworm data was obtained from http://www.silkdb.org.

Comparison of the transcriptome pattern of *P. xylostella *for eight different GO terms (molecular function - Level 2) with those of silkworm http://www.silkdb.org showed high similarity in the distribution of genes across GO categories indicating that the transcriptome analysed was not biased towards particular categories (Figure 3).

Overall, from the assembled contigs of over 200 bp in length, 44% showed similarity with genes or proteins in the NCBI database. The rest may represent unknown genes, non-coding RNA or misassembled contigs that are expected due to the presence of large repetitive or duplicated regions. David *et al*. (2010) suggested that a significant proportion of transcript signatures detected outside predicted genes represent regulatory non-coding RNAs, because these large numbers of non-coding RNA can be antisense, intergenic or overlapping with protein-coding genes [22].

### Effects of parasitism on the transcription of host immune-related genes

**Figure 4:**
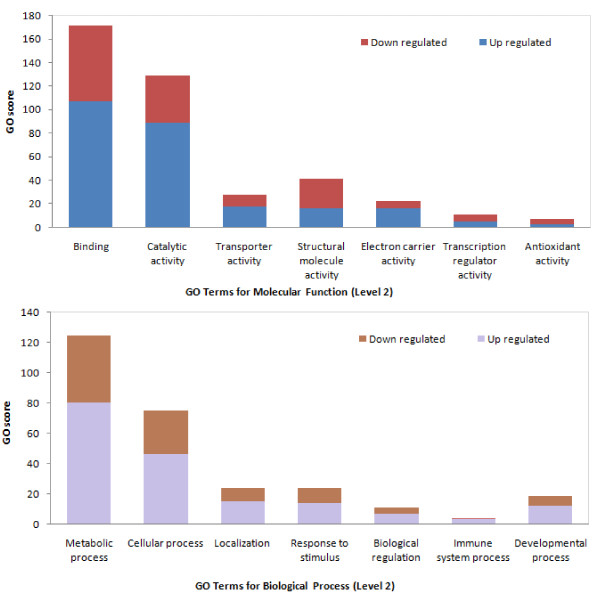
**The gene ontology score for some selected GO terms (level 2) for significantly up- and down-regulated transcripts**. (A) Molecular Function (B) Biological Process. Binding and catalytic activity had the highest GO scores for Molecular Function among those genes that were differentially expressed after parasitization. In the Biological Process category, two GO terms of metabolic and cellular processes showed the highest GO scores. In each group, the abundance of up-regulated genes was higher than down-regulated genes.

Our sequencing data analyses indicate that parasitism has a significant impact on the transcriptome profile of *P. xylostella *larvae. The 'Oases' package was used to assemble data for differential expression analysis. Initially, reads from the parasitized and non-parasitized larvae were cleaned and combined, before *de novo *transcriptome assembly was carried out using Oases 0.1.18. [32]. The individual sets of reads were then mapped back to the previously assembled contigs and counted as a proxy for gene expression. After filtering our dataset using criteria such as number of reads, contig length, E-value (for nearest homolog identity) and greater than 2-fold change, 928 contigs were short-listed. Figure 4 shows that most of the differentially expressed transcripts for the selected GO terms (Level 2), molecular function and biological process, were up-regulated. Among the contigs, only those related to immunity and development, which were differentially transcribed after *D. semiclausum *parasitization, are displayed in Tables 2 and 3, respectively, since these are the genes most relevant to parasitism. In instances where differential expression of *P. xylostella *genes following *D. semiclausum *parasitization may not be consistent with proteomic observations in other host-parasitoid systems previously reported (e.g. [2]), it is possible that post-transcriptional inhibitory effects of parasitoids' maternal factors (e.g. PDVs or venom) contribute to these differences. It has been reported that PDV genes are able to interrupt translation of host genes with their host translation inhibitory factors (HTIF) which was initially characterized from *Campoletis sonorensis *IV (*Cs*IV) [33]. Accordingly, in *Heliothis virescens *it was shown that lysozyme activity declined after parasitization by *C. sonorensis *or injection of *Cs*IV; however, the transcript levels of the gene increased after parasitization. This suggested that *Cs*IV may regulate host cell gene expression at the translation level. We also found that lysozyme transcript levels increased following parasitization (Table 2). Another study also showed that lysozyme concentration and activity in *P. xylostella *larvae parasitized by *Cotesia plutellae *was decreased [2]; however, transcript levels were not measured. Recently, Barandoc and Kim [34] showed that the translation of storage protein in *P. xylostella *was inhibited by two BV genes (CpBV15α and CpBV15β). In another study, it was shown that expression of antimicrobial peptides (diptericin, cecropin A and drosomycin) were either unchanged or minimally induced in parasitized *Drosophila melanogaster *larvae by *Leptopilina boulardi *[35]. The authors concluded that antimicrobial genes are regulated differently independent of those mediating cellular encapsulation.

**Table 2:** A list of *P .xylostella *immune-related genes that were differentially transcribed after parasitization by *D. semiclausum*

Sequence ID	**TSA Accession No**.	Nt. Length	Fold change	Protein	Species	E value	Nt. ID (%)	**Accession no**.
Locus 2992	JL943792	517	11	Moricin-like peptide C2	*Galleria mellonella*	5.00E-12	75	ABQ42576.1
Locus 11864	JL943746	452	7.06	Serine proteinase inhibitor	*Procambarus clarkii*	5.00E-14	41	AAQ22771.1
Locus 13987	JL943753	281	6.97	Gloverin	*Plutella xylostella*	3.00E-49	100	ACM69342.1
Locus 42625	JL943821	487	6.71	Beta-1,3-glucan recognition protein 3	*Helicoverpa armigera*	5.00E-30	45	ACI32828.1
Locus 652	JL943855	803	6.25	Proline-rich protein	*Galleria mellonella*	6.00E-18	38	ACQ99193.1
Locus 10530	JL943744	1440	6.09	Transferrin	*Plutella xylostella*	0	99	BAF36818.1
Locus 19881	JL943772	503	4.96	Odorant binding protein	*Heliothis virescens*	1.00E-49	66	ACX53743.1
Locus 8118	JL943865	992	4.51	Serine proteinase	*Samia cynthia ricini*	6.00E-89	55	BAF43531.1
Locus 4070	JL943818	449	4.37	Cecropin E	*Plutella xylostella*	8.00E-17	100	BAF36816.1
Locus 6056	JL943850	650	4.3	Cecropin 1 (antibacterial peptide)	*Plutella xylostella*	1.00E-16	98	ADA13281.1
Locus 2740	JL943785	349	4.23	Thrombin inhibitor infestin	*Triatoma infestans*	7.00E-19	47	AAK57342.1
Locus 742	JL943860	1405	3.99	Lysozyme II	*Artogeia rapae*	4.00E-15	76	AAT94286.1
Locus 8845	JL943866	517	3.81	Peptidoglycan recognition protein	*Plutella xylostella*	2.00E-86	97	BAF36823.1
Locus 3377	JL943803	744	3.78	Peptidoglycan recognition protein S6	*Bombyx mori*	3.00E-58	61	NP_001036858.1
Locus 5206	JL943842	712	3.67	Hemolin	*Plutella xylostella*	7.00E-131	97	ACN69054.1
Locus 11896	JL943747	1453	3.28	Prophenoloxidase-activating proteinase 3	*Plutella xylostella*	0	93	BAF36824.1
Locus 4541	JL943828	843	3	Trypsin-like serine proteinase 1	*Plutella xylostella*	2.00E-37	42	ADK66277.1
Locus 3869	JL943816	1604	2.91	Bifunctional protein folD	*Culex quinquefasciatus*	2.00E-101	63	XP_001846734.1
Locus 30146	JL943795	236	2.91	Nucleotide excision repair protein	*Bombyx mori*	1.00E-28	71	NP_001177140.1
Locus 14548	JL943754	580	2.87	Beta-1,3-glucan recognition protein 2	*Bombyx mori*	5.00E-40	49	NP_001037450.1
Locus 15717	JL943757	333	2.73	Prophenoloxidase activating factor 3	*Bombyx mori*	3.00E-19	57	AAL31707.1
Locus 13921	JL943752	602	2.68	Serine protease 33	*Mamestra configurata*	2.00E-36	50	ACR15983.2
Locus 12724	JL943748	1288	2.51	Serine protease inhibitor 7	*Bombyx mori*	5.00E-94	46	NP_001139701.1
Locus 43519	JL943823	256	2.5	Ceramidase	*Aedes aegypti*	2.00E-21	62	XP_001658093.1
Locus 48995	JL943836	207	2.5	Toll receptor	*Tribolium castaneum*	2.00E-19	59	XP_971999.1
Locus 1006	JL943743	1154	2.41	Lipocalin	*Bombus ignitus*	1.00E-89	64	ADA82597.1
Locus 6114	JL943851	1293	2.38	Lysosomal acid lipase	*Tribolium castaneum*	1.00E-65	39	XP_972957.2
Locus 28994	JL943788	911	2.33	Pattern recognition serine proteinase	*Manduca sexta*	3.00E-87	51	AAR29602.1
Locus 20345	JL943774	322	2.26	Trypsin T6	*Heliothis virescens*	9 E-16	54	ABR88249.1
Locus 17873	JL943765	412	2.25	NADP+	*Danio rerio*	1.00E-56	76	NP_998058.1
Locus 7994	JL943864	1305	2.24	Serine protease inhibitor (pxSerpin 3)	*Plutella xylostella*	2.00E-99	50	BAF36821.1
Locus 2093	JL943776	2890	2.21	Apolipophorins	*Manduca sexta*	0	67	Q25490.1
Locus 20404	JL943775	251	2.19	Serine protease inhibitor (Serpin 13)	*Bombyx mori*	4.00E-14	49	NP_001139705.1
Locus 41604	JL943819	282	2.16	Broad-Complex isoform Z2	*Bombyx mori*	1.00E-30	98	BAD24051.1
Locus 35475	JL943806	363	2.15	Heat Shock Protein (HSP70)	*Acyrthosiphon pisum*	3.00E-12	44	XP_001950064.1
Locus 29819	JL943791	461	2.14	Beta-1,3-glucan recognition protein 2a	*Helicoverpa armigera*	1.00E-53	64	ACI32826.1
Locus 4518	JL943827	303	2.13	Serine protease 1	*Lonomia obliqua*	4.00E-22	51	AAV91432.2
Locus 39662	JL943817	299	2.12	1-phosphatidylinositol-4,5-bisphosphate	*Bombyx mori*	6.00E-45	86	NP_001165393.1
Locus 9824	JL943870	627	2.11	Peripheral-type benzodiazepine receptor	*Bombyx mori*	6.00E-34	57	NP_001040343.1
Locus 19848	JL943771	1136	2.1	Peroxidasin	*Tribolium castaneum*	1.00E-134	62	XP_968570.1
Locus 21658	JL943781	437	2.04	Hemolymph proteinase 8	*Manduca sexta*	4 E-49	71	AAV91006.1
Locus 620	JL943852	1032	-2	Heat Shock Protein (HSP90)	*Plutella xylostella*	8.00E-140	100	BAE48742.1
Locus 18755	JL943766	351	-2.06	Vasorin	*Culex quinquefasciatus*	1.00E-54	85	XP_001870087.1
Locus 15108	JL943755	615	-2.07	Arylalkylamine N-acetyltransferase	*Antheraea pernyi*	1.00E-29	38	ABD17803.1
Locus 19225	JL943768	253	-2.12	Flap endonuclease	*Carukia barnesi*	6.00E-25	83	ACY74444.1
Locus 29249	JL943789	213	-2.12	Putative lysozyme	*Bombyx mori*	4.00E-22	67	ADA67927.1
Locus 7618	JL943861	3056	-2.14	Fascin	*Tribolium castaneum*	0	79	XP_972494.1
Locus 13139	JL943751	701	-2.4	Hemocyte protease-1	*Bombyx mori*	6 E-75	60	BAG70409.1
Locus 11481	JL943873	364	-3.25	Trypsin	*Helicoverpa armigera*	5 E-16	43	ACB54939.1
Locus 24418	JL943783	211	-3.5	Black (DOPA-deC-like)	*Papilio xuthus*	1.00E-29	87	BAI87832.1
Locus 536	JL943843	527	-3.65	Catalase	*Takifugu obscurus*	1.00E-17	35	ABV24056.1
Locus 3343	JL943800	289	-3.78	Immune-related Hdd1	*Hyphantria cunea*	3.00E-11	39	AAD09279.1
Locus 13049	JL943750	452	-5.81	Putative defense protein Hdd11	*Hyphantria cunea*	8.00E-55	67	O96382.1

Fold changes were generated by comparing average read depths for each contig.

**Table 3:** Developmental- and non-immune metabolism-related transcripts of *P. xylostella*, which were differentially expressed after *D. semiclausum *parasitization

Sequence ID	**TSA Accession No**.	Nt. Length	Fold change	Protein	*Species*	E value	Nt. ID (%)	**Accession no**.
Locus 29717	JL943790	504	27.86	Endonuclease-reverse transcriptase	*Bombyx mori*	1 E-46	67	ADI61832.1
Locus 3567	JL943808	277	20.01	Glucose dehydrogenase precursor	*Pediculus humanus corporis*	1 E-15	52	XP_002429706.1
Locus 784	JL943863	1943	12.5	Methionine-rich storage protein 1	*Plutella xylostella*	0	100	BAF45385.1
Locus 50007	JL943839	234	8.31	Putative RecQ Helicase	*Heliconius melpomene*	1 E-11	63	CBH09254.1
Locus 3712	JL943813	1744	7.8	Methionine-rich storage protein 2	*Plutella xylostella*	0	100	BAF45386.1
Locus 1098	JL943745	724	4.4	Arylphorin-like hexamerin-2	*Plutella xylostella*	1 E-141	100	BAF32562.1
Locus 9922	JL943871	1790	4.31	44 kDa zymogen (serine protease)	*Tenebrio molitor*	9 E-61	35	BAG14262.1
Locus 2239	JL943782	2186	3.46	Methionine-rich storage protein	*Spodoptera exigua*	0	59	ABX55887.1
Locus 352	JL943805	1023	3.43	Arylphorin-like hexamerin-1	*Plutella xylostella*	2 E-172	89	BAF32561.1
Locus 5917	JL943849	2338	3.4	Phenylalanine hydroxylase	*Papilio xuthus*	0	88	BAE66652.1
Locus 42829	JL943822	273	3.33	Syntaxin	*Culex quinquefasciatus*	3 E-21	60	XP_001865470.1
Locus 1575	JL943758	1738	3.12	Phosphoribosylaminoimidazole carboxylase	*Bombyx mori*	0	90	NP_001040376.1
Locus 33387	JL943799	375	3.11	Insulin receptor	*Bombyx mori*	3 E-34	59	NP_001037011.1
Locus 47369	JL943832	347	3	Leucine-rich transmembrane protein	*Pediculus humanus corporis*	1 E-24	60	XP_002422869.1
Locus 7126	JL943859	1075	2.98	Sugar transporter 4	*Bombyx mori*	2 E-104	64	NP_001165395.1
Locus 35539	JL943807	592	2.55	Torso-like protein	*Tribolium castaneum*	2 E-31	38	NP_001107843.1
Locus 36082	JL943809	735	2.44	Reverse transcriptase	*Aedes aegypti*	3 E-36	39	AAZ15237.1
Locus 199	JL943773	1588	2.4	S-adenosyl-L-homocysteine hydrolase	*Plutella xylostella*	0	100	BAF36817.1
Locus 5556	JL943845	1186	2.33	Yellow-fa	*Bombyx mori*	4 E-141	69	NP_001037424.1
Locus 4588	JL943829	205	2.24	Endoprotease FURIN	*Spodoptera frugiperda*	4 E-28	87	CAA93116.1
Locus 4894	JL943835	861	2.24	Lipase	*Helicoverpa armigera*	3 E-50	54	ACB54943.1
Locus 4689	JL943830	2429	2.21	Cathepsin L precursor	*Tribolium castaneum*	0	62	NP_001164088.1
Locus 3008	JL943793	950	2.21	Hemolymph proteinase 5	*Manduca sexta*	2 E-101	62	AAV91003.1
Locus 20931	JL943777	661	2.17	Gamma-glutamyl transferase	*Bombyx mori*	1 E-62	56	NP_001165385.1
Locus 21405	JL943780	705	2.14	Juvenile hormone binding protein	*Manduca sexta*	2 E-27	35	AAB25736.2
Locus 3797	JL943815	1653	2.13	Imaginal disk growth factor	*Plutella xylostella*	0	100	BAF36822.1
Locus 5088	JL943841	1476	2.12	Cathepsin B-like cysteine proteinase	*Spodoptera exigua*	1 E-146	81	ABK90823.1
Locus 1887	JL943767	1872	2.1	Cathepsin D isoform 1	*Tribolium castaneum*	7 E-165	73	XP_966517.1
Locus 47019	JL943831	207	2.09	DNA-binding protein Ewg putative	*Pediculus humanus corporis*	3 E-22	93	XP_002430412.1
Locus 16109	JL943872	515	2.08	Nesprin-1	*Pediculus humanus corporis*	3 E-26	36	XP_002427810.1
Locus 41758	JL943820	222	2.06	Neuroglian	*Mythimna separata*	4 E-29	78	BAI49425.1
Locus 6830	JL943856	427	2.04	Cytochrome P450	*Plutella xylostella*	2 E-67	96	ABW34440.1
Locus 17556	JL943762	1272	2.03	Arginase	*Bombyx mori*	9 E-144	72	BAH19308.1
Locus 25700	JL943784	286	-2.03	Cytochrome P450 monooxygenase	*Helicoverpa zea*	7 E-16	74	AAM54723.1
Locus 7042	JL943858	809	-2.05	Tyrosine transporter	*Aedes aegypti*	2 E-18	76	XP_001658764.1
Locus 4738	JL943833	1359	-2.07	Collagen	*Bombyx mori*	2 E-23	49	CAA83002.1
Locus 3369	JL943802	471	-2.09	Trypsin alkaline B	*Manduca sexta*	7 E-37	71	P35046.1
Locus 7004	JL943857	696	-2.13	Cuticular protein glycine-rich 20	*Bombyx mori*	3 E-15	52	NP_001166784.1
Locus 17043	JL943761	237	-2.13	Voltage & ligand gated potassium channel	*Culex quinquefasciatus*	2 E-15	71	XP_001853758.1
Locus 16717	JL943760	1585	-2.14	Multidrug resistance protein 2	*Culex quinquefasciatus*	1 E-93	41	XP_001866984.1
Locus 5571	JL943846	389	-2.19	Sugar transporter	*Aedes aegypti*	1 E-11	48	XP_001652873.1
Locus 13022	JL943749	216	-2.36	Ecdysis-triggering hormone	*Manduca sexta*	2 E-15	65	AAD45613.1
Locus 6441	JL943853	661	-2.43	Retinol dehydratase	*Spodoptera frugiperda*	1 E-53	58	AAC47136.1
Locus 15939	JL943759	279	-2.46	Cyclin-dependent kinase 2-like	*Saccoglossus kowalevskii*	3 E-23	68	XP_002740677.1
Locus 487	JL943834	1615	-2.56	Glucosinolate sulphatase	*Plutella xylostella*	0	64	CAD33828.1
Locus 6512	JL943854	484	-2.57	Phosphohistidine phosphatase	*Bombyx mori*	3 E-45	62	NP_001040265.1
Locus 7636	JL943862	454	-2.7	Reverse transcriptase	*Ostrinia nubilalis*	3 E-39	48	ABO45231.1
Locus 33235	JL943798	220	-2.74	Juvenile hormone epoxide hydrolase	*Bombyx mori*	5 E-18	64	NP_001159617.1
Locus 36575	JL943812	209	-2.83	Lipase	*Bombyx mori*	8 E-12	52	ADA67928.1
Locus 5577	JL943847	1813	-2.9	Chitinase	*Plutella xylostella*	0	100	ACU42267.1
Locus 4357	JL943824	1009	-3.21	Cuticular protein RR-1 motif 10	*Bombyx mori*	1 E-56	68	NP_001166738.1
Locus 21088	JL943778	247	-3.25	Carboxypeptidase M	*Aedes aegypti*	1 E-37	83	XP_001661307.1
Locus 2898	JL943787	485	-4.27	Acheron	*Manduca sexta*	1 E-68	88	AF443827_1
Locus 5445	JL943844	1708	-8.82	Urbain	*Bombyx mori*	4 E-24	37	NP_001139414.1
Locus 21299	JL943779	290	-15.5	37-kDa serine protease	*Bombyx mori*	6 E-41	81	NP_001128675.1
Locus 17795	JL943764	363	-24.01	Cuticle protein	*Aedes aegypti*	3 E-29	67.78	XP_001659461.1
Locus 8908	JL943867	625	-26.71	Ecdysteroid regulated protein	*Manduca sexta*	1 E-12	71	AAA29312.1

Fold changes were generated by comparing average read depths for each contig.

In addition to post-transcriptional effects of PDV infection, there may also be variations in different host-parasitoid interactions with regard to host transcripts affected by parasitization/PDV infection. In a very recent study, it was shown that in *Spodoptera frugiperda *larvae injected with *Hyposoter didymator *ichnovirus (*Hd*IV) or *Microplitis demolitor *bracovirus (*Md*BV), the differentially expressed transcripts in hemocytes and fat body largely differed depending on the PDV injected suggesting that the tissues responded differently to the different viruses [36]. However, there were a number of host genes that responded similarly when infected with *Hd*IV or *Md*BV. Based on this study, *Hd*IV affected transcript levels in both hemocytes and fat body, whereas *Md*BV mostly affected gene expression in the fat body.

**Figure 5:**
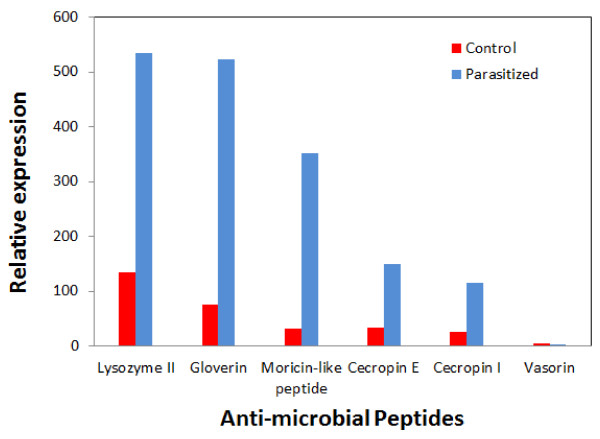
**Relative gene expression values based on average read depth for selected antimicrobial peptide classes in non-parasitized (control) and parasitized *P. xylostella *larvae**.

In our study, we found that antimicrobial peptides such as gloverin, moricin, lysozyme II and cecropin were up-regulated after parasitoid attack, compared to transcription levels in control unparasitized larvae. Among these, lysozyme II and gloverin had the highest transcription levels in parasitized *P. xylostella *larvae relative to other antimicrobial peptides (Figure 5; Table 2). Transcription levels for cecropin 1 and pxCecropin E increased by more than 3-fold after *D. semiclausum *attack (Table 2). It has been reported that immune-related genes were also up-regulated in *P. xylostella *larvae in response to microbial challenge (e.g. pxCecropin 5.94 fold, hemolin 3.18 fold and cecropin E 4.99 fold) [37]. Barandoc *et al*. measured pxCecropin expression levels by quantitative RT-PCR (qRT-PCR) in parasitized *P. xylostella *larvae and showed that pxCecropin was suppressed by *C. plutellae *BV [12]. Some antimicrobial peptides, such as moricin and gloverin, were previously found not to be induced by bacterial challenge of lepidopteran larvae, while lysozyme and cecropins are well-known inducible antimicrobial peptides [12,38]. Recently, it has been reported that gloverin expression was changed after bacterial infection in *P. xylostella *[39]. Our data shows that immune responses after parasitoid attack appear to be different from microbial challenge responses, because gloverin and moricin-like peptide were up-regulated about 7 and 11 fold, respectively, after *D. semiclausum *attack.

Other related genes which were up-regulated in parasitized larvae included a serine protease and serine protease inhibitor (pxSerpin 3) (Table 2). Serine proteases are major immune regulatory proteins, which are found in a wide range of species from insects to mammals. In contrast, a proteomics analysis showed that pxSerpin 2 was suppressed in *P. xylostella *larval plasma during parasitism by *C. plutellae *[7]. Beck *et al*. demonstrated that the ovarian calyx fluid of the ichneumonid endoparasitoid *Venturia canescens *has the potential to suppress the host immune system due to a putative serpin activity [40]. Here, we found that serine protease inhibitors (JL943746, JL943748, JL943775, JL943864) were over-expressed two- to seven-fold, after parasitism in *P. xylostella*. Thus, the induction of serine protease inhibitors upon immune challenge in parasitized larvae could be part of an endoparasitoid immune suppressive strategy. Many insect protease inhibitors are known to inactivate enzymes isolated from entomopathogenic fungi, and their involvement in insect-pathogen interactions has been widely postulated [41]. Aguilar *et al*. reported that five serine proteases involved in a single metabolic cascade were up-regulated in *Anopheles gambiae *upon microbial challenge, but they suggested a role for the proteases in protecting the mosquito from detrimental effects of an uncontrolled spread of immune reaction [42].

Serine proteases also play a significant role in the activation of the prophenoloxidase (proPO) cascade. In insects, proPO is activated upon injury or invasion, which results in localized melanization of the wound area and/or melanotic capsules capturing invading microorganisms and parasites [43]. In the current study, proPO activating protease (PAP) transcription was up-regulated in parasitized larvae of *P. xylostella*. In a genome-wide microarray study of *D. melanogaster*, several genes encoding enzymes of the melanization cascade were found to be up-regulated by *L. boulardi *parasitization [44], consistent with this study. Asgari *et al*. (2003) reported that venom protein (Vn50) from *Cotesia rubecula *is homologous to serine protease homologs [43]. It is likely that the injection of this protein (or putative homologs thereof) by parasitoid wasps into the host body, may interfere with the proteolytic cascade that leads to the activation of proPO. cDNA microarray analysis of *S. frugiperda *hemocytes and fat body 24 hours after injection of *Hd*IV revealed differential expression of several host genes [45]. Among these, eight immune-related genes showed differential expression in hemocytes with proPO-1 and proPO-2 showing up-regulation, while PAP transcript levels declined. Other immune-related genes that were differentially expressed in the hemocytes were galectin, which showed up-regulation, whereas scavenger R, immulectin-2, lysozyme and calreticulin showed down-regulation [45]. A recent study confirmed up-regulation of proPOs in *S. frugiperda *larvae injected with *Hd*IV; however, in the same host injected with *Md*BV, proPO transcript levels declined [36] which suggested differential responses of the host to different PDVs.

Transcripts of most proteins, which are involved in the Toll pathway such as Relish, Dorsal, Pelle, Cactus and Toll receptor, were found in our deep sequencing analysis, but only transcription levels of proteins that showed similarity to the Toll receptor were up-regulated (2.5 fold; Table 2). In *D. melanogaster *larvae parasitized by *Asobara tabida *or *L. boulardi*, components of the Toll/Imd (Immune deficiency) pathways were up-regulated, and antimicrobial peptide expression was increased [44,46]. In addition, in *Drosophila*, Toll and Imd pathways are required for activation and stimulation of NF-κBs signal transduction and also responsible for innate immune response in parasitized *Drosophila *[47]. NF-κB proteins are a family of proteins in eukaryotes that are involved in the control of a large number of cellular and organismal processes, such as immune responses, developmental processes, cellular growth, and apoptosis. Furthermore, NF-κB signalling is important in immune inducibility of pathogen-associated-molecular-patterns, and it is widely assumed that it plays a conserved role in invertebrate immune regulation [48,49].

It has been suggested that PDV-expressed vankyrin proteins may interfere with NF-κB-mediated signalling during immune response and development in parasitized larvae [15,50]. Fath-Goodin *et al*. (2009) reported that *Cs*IV vankyrin genes also encode proteins with sequence homology to the inhibitory domains of NF-κB transcription factor inhibitors [13].

The results of our transcriptome analysis also indicated that genes known to be involved in insecticide resistance/detoxification are up-regulated following parasitism (Table 2). In agreement, it has previously been reported that cytochrome P450 (CYP) and glutathione-S-transferase (GST) activities increased in parasitized *P. xylostella *larvae [29]. Takeda et al. (2006) suggested that parasitoid larvae contributed to CYP activity enhancement since the parasitoid hatched two days after oviposition and the CYP activity was significantly increased three days after parasitization [29].

Our analyses also detected a considerable number of other immune-related genes whose transcription levels altered after parasitization by *D. semiclausum*. However, only a small group affected by parasitoid attack were found to be altered enough to be statistically biologically relevant (i.e. showing greater than two-fold change).

### Transcription levels of host development-related genes

Generally, larval endoparasitoids lay their eggs into the host hemocoel, and their progenies develop by consuming host hemolymph and tissues. As a consequence, the parasitoid's larval growth also fully depends on the host's development [1,51,52]. In *P. xylostella *larvae parasitized by *D. semiclausum*, development is arrested at the prepupal stage [53]. Developmental arrest before pupation is one of the most common effects of PDVs and/or other maternal factors injected by many endoparasitoids into their hosts [14,54-56]. In these interactions, the parasitoid larvae and PDVs are responsible for increasing the juvenile hormone (JH) titre in host larvae and preventing ecdysteroid levels from rising sufficiently to allow host pupation [57-61].

Based on our transcriptome data, parasitism by *D. semiclausum *leads to down-regulation of genes associated with ecdysteroid activities; for example, the transcription level of ecdysteroid regulated protein was down-regulated more than 26 times in parasitized larvae (Table 3). Considering that ecdysteroids are required to trigger expression of ecdysteroid regulated protein [62], and that parasitism in general leads to reductions in ecdysteroid titres [54,63-66], down-regulation of ecdysteroid regulated protein is expected.

Juvenile hormone binding protein (JHBP) may protect JH from non-specific degradation and adsorption by preventing exposure of JH to epoxide hydration by JH epoxide hydrolase (JHEH), which generates the hormonally inactive JH diol [67]. In agreement, JHBP transcript levels were up-regulated more than 2 times and interestingly, transcription levels of JHEH were down-regulated more than 2 times (Table 3). In all the reported host-parasitoid systems, it seems that JH is maintained at high levels during parasitoid larval development [58,66,68]. Therefore, an increase in JHBP, and corresponding decrease in JHEH transcription levels, after parasitism, seems logical to maintain higher JH levels during parasitism.

As indicated above, previous studies have shown that PDVs may inhibit translation of specific storage or growth-associated proteins despite up-regulation (or steady-state) of transcript levels of the encoding genes, following parasitism or injection of PDVs [69-71]. In this study, we found that arylphorin and methionine-rich storage proteins were over-transcribed in parasitized larvae (Table 3); however, their translation may be affected similarly to mechanisms in the reports discussed above, which needs to be experimentally shown.

#### Quantitative RT-PCR validation of transcriptome analysis

**Figure 6:**
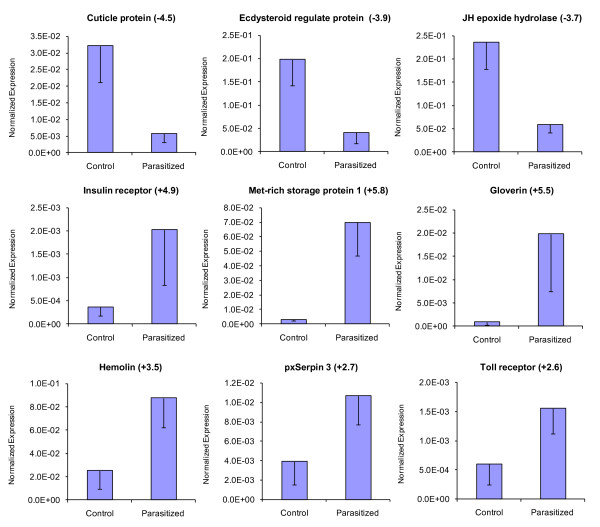
**qRT-PCR analysis of nine selected genes from *P. xylostella *which showed differential expression after parasitization based on deep sequencing analysis**. Error bars indicate standard deviations of averages from three replicates. Fold changes are shown in brackets.

To validate our deep sequencing data, nine differentially regulated *P. xylostella *genes were selected from immune- and development-related genes (Tables 2 and 3) for qRT-PCR analysis, using the same RNA samples as for deep sequencing. These were cuticle protein, ecdysteroid regulated protein, JH epoxide hydrolase, insulin receptor, methionine-rich storage protein 1, gloverin, hemolin, pxSerpin 3 and Toll receptor. The qRT-PCR results confirmed the data obtained from deep sequencing analysis showing similar trends in up- or down-regulation of host genes (Figure 6). For example, based on deep sequencing analysis, gloverin, Toll receptor, and pxSerpin 3 were up-regulated 6.9, 2.5 and 2.2 fold, respectively (Table 2), and showed 5.5, 2.6 and 2.7 fold changes, respectively in qRT-PCR analyses (Figure 6).

**Figure 7:**
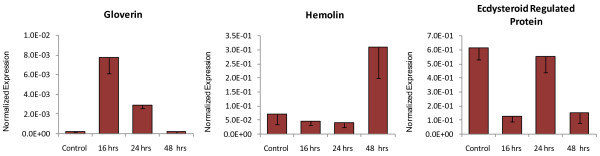
**qRT-PCR analysis of expression levels of three selected genes in 2^nd ^instar *P. xylostella *larvae at three time points after parasitization with *D. semiclausum*, which showed differential expression after parasitization based on deep sequencing analysis**. Error bars indicate standard deviations of averages from three replicates.

Since the samples analysed above were pools of RNA from various time points, and it is known that expression of host genes may vary at different periods after parasitization, we further isolated RNA from 2^nd ^instar *P. xylostella *larvae at 16, 24 and 48 hrs after parasitization, and analyzed three associated genes by qRT-PCR. These were hemolin, gloverin and the ecdysteroid regulated protein. For each time point, a mixture of 10 larvae was used. As expected, there were fluctuations in the expression levels of these genes following parasitization (Figure 7). For example, gloverin expression was highly induced at 16 hrs after parasitization, subsequently declined at 24 hrs, and then further reduced to the same level as unparasitized larvae at 48 hrs post-parasitization (Figure 7). Hemolin was only up-regulated at 48 hrs after parasitization. Expression of ecdysteroid regulated protein was initially down-regulated at 16 hrs after parasitization, but increased at 24 hrs before subsequently declining by 48 hrs post-parasitization (Figure 7).

### *Diadegma semiclausum* ichnovirus genes

PDV genes are divided into three groups based on whether they are expressed in the carrier wasp (class I), the infected host larvae (class II) or both (class III) [72]. Among these genes, class II genes have received the greatest attention and have been studied more than other groups [73]. The genomes of some IVs, such as those found in the wasps *C. sonorensis, C. chlorideae, Hyposoter fugitivus, H. didymator, and Tranosema rostrale*, have been sequenced and resultant data are available on public databases [21,72,74]. Six conserved gene families: repeat element, cysteine motif, viral innexin, viral ankyrin, N-family and the polar-residue-rich proteins (a newly defined putative family), have been reported in most IV genomes [72].

Here, expression of a range of *Ds*IV genes were detected in parasitized larvae. In our analysis, 19 unique sequences were identified from five PDV gene families including vankyrin, viral innexin, repeat elements, cysteine-rich motif, and polar residue rich protein families (Table 4). In addition, five other putative virus protein sequences with unknown function were identified in parasitized larvae, and showed more than 50% similarity with some parts of IV reference genomes, but no putative specific protein domain homologies were detected in their sequences (Table 4). The online open reading frame finder tool at the NCBI website http://www.ncbi.nlm.nih.gov/projects/gorf was used for prediction of full-length sequences in *Ds*IV genes and only four of these genes are reported here as full-length (Table 4).

**Table 4:** *D. semiclausum *IV transcripts which were detected in parasitized *P. xylostella *larvae

Protein	Length (nt)	Accession Number	Similarity (Protein/virus/Accession No.)	length (aa)	E value	Nt. ID %	Conserved Domains
Vankyrin 1	671*	JI257593	vankyrin-b17 (HfIV) AAS90270.1	170	3.56E-52	61	Yes
Vankyrin 2	519*	JI257594	vankyrin-d8.3 (HfIV) BAF45734.1	159	6 E-64	80	Yes
Vankyrin 3	257	JI257595	hypothetical protein 2 (HdIV) AAR99845.1	126	3.58E-23	61	Yes
Vankyrin 4	361	JI257596	vankyrin-b1(HfIV) AAX24120.1	167	2 E-39	76	Yes
Viral Innexin 1	1370	JI257597	viral innexin-b5.1 (HfIV) BAF45654.1	354	5 E-95	60	Yes
Viral Innexin 2	576	JI257598	viral innexin-c16 (HfIV) AAS58041.1	371	2 E-81	75	Yes
Repeat element 1	833	JI257599	repeat element protein 7 (HdIV) AAR89179.1	224	4.32E-73	67	Yes
Repeat element 2	697	JI257600	repeat element protein-d10.1 (HfIV) BAF45740.1	244	1.53E-72	64	Yes
Repeat element 3	947	JI257601	repeat element protein-c18.1 (HfIV) BAF45697.1	244	9.52E-94	71	Yes
Repeat element 4	859*	JI257602	repeat element protein-e2.3 (HfIV) BAF45758.1	209	3.40E-76	67	Yes
Repeat element 5	656	JI257603	repeat element protein (HdIV) AAO16959.1	225	1.21E-73	59	Yes
Repeat element 6	500	JI257604	repeat element protein-d11.2 (HfIV) BAF45744.1	244	1E-68	72	Yes
Cysteine rich motif	630	JI257605	cysteine motif gene-d9.1(HfIV) BAF45736.1	311	2.99E-55	64	Yes
Polar residue rich protein	671	JI257606	polar residue rich protein-b13.2 (HfIV) BAF45664.1	159	1.24E-12	42	No
Unknown Protein	256	JI257607	c7-2.1 (TrIV) BAF45599.1	119	2.90E-17	68	Yes
Unknown Protein	2143*	JI257608	c12.1 (HfIV) AAS68099.1	432	2E-107	50	No
Unknown Protein	418	JI257609	P12 (HdIV) AAS83461.1	106	6E-18	58	No
Unknown Protein	584	JI257610	c10.1 (HfIV) AAS90272.1	385	9.15E-50	51	No
Unknown Protein	208	JI257611	b5.3 (HfIV) BAF45655.1	115	9.57E-13	69	No

* Full length

Hf: *Hyposoter fugitives*

Hd: *Hyposoter didymator*

Tr: *Tranosema rostrale*

**Figure 8:**
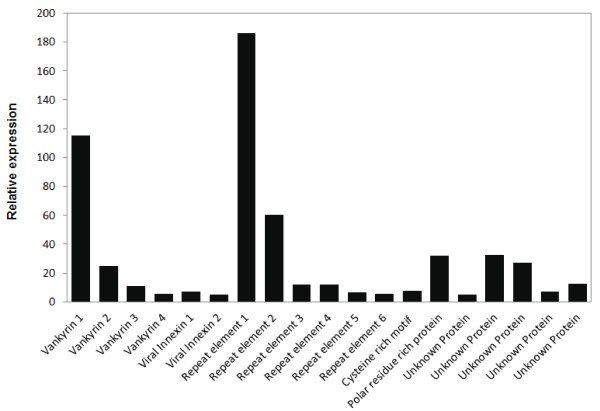
**Relative gene expression values based on average read depth for all detected *D. semiclausum *ichnovirus genes**. RPKM normalized values were used to generate the data.

Presence of multiple sequences for each PDV gene family is common in other IVs and we identified four sequences with vankyrin domain and two viral innexin transcripts, which showed high similarity with *H. fugitivus *IV innexins (Table 4). Repeat element domains were also identified in six sequences with translated lengths of between 209-244 amino acids. Transcription levels (or gene expression values) were found to be different among different members within each gene family. Vankyrin 1 and repeat element 1 had the highest transcription levels in their respective families, and also relative to other *Ds*IV genes (Figure 8).

**Figure 9:**
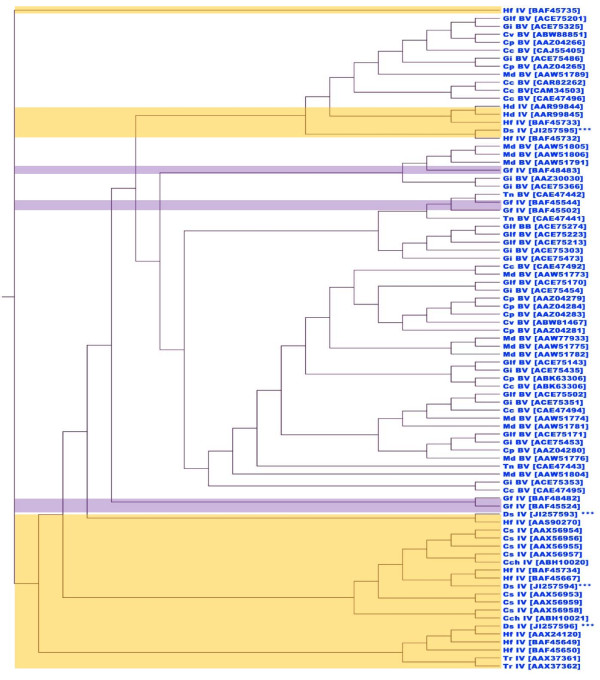
**Hierarchical Cluster analysis of vankyrin gene sequences of all reported PDV vankyrins, using neighbour-joining algorithm**. The accession code for each vankyrin is provided in brackets. The yellow and purple backgrounds show the *Ichnovirus *and provisional group of banchine virus genes, respectively, and the rest are *Bracovirus *vankyrins.

Hierarchical cluster analysis of vankyrin gene sequences of all reported PDV vankyrins (protein sequences at NCBI) using a neighbour-joining algorithm, classified BVs and IVs into two major groups (Figure 9). All four *Ds*IV vankyrins that were identified in this study have high similarities with other IVs and were distributed into four separate clusters indicating that these members of the ankyrin family possibly originated from different segments of the *Ds*IV genome or at least there has been some ancient gene duplication and/or differential selection even if encoded on the same circle. In addition, the separate clustering of vankyrin genes suggests that they are not closely related, and therefore did not likely undergo recent duplication to form similar paralogs.

## Conclusion

Overall, this study provides the first comprehensive analysis of the impact of a parasitoid wasp on its host at the transcriptomic level, using RNA deep sequencing technique. The results showed differential expression of a large number of *P. xylostella *genes, including immune-related genes, upon parasitization by *D. semiclausum*. In addition, although presence of *Ds*IV particles has been reported in parasitized larvae, our results provide evidence for expression of 19 *Ds*IV genes expressed in the host, which have not been previously reported. Analysis of these sequences indicated the presence of conserved genes that belong to major IV class II genes. The transcriptome profiling data sets obtained in this study provide a basis for future research in this under-explored host-parasitoid interaction. In addition, the identified immune-, development- and detoxification-related genes may be targets for *P. xylostella *control and allow manipulation of host-parasite interactions.

## Methods

### Insects and parasitization

*P. xylostella *and the parasitoid wasp (*D. semiclausum*) were raised on cabbage plants and host larvae, respectively, at 25°C. Twenty five 3^rd ^and 4^th ^instar *P. xylostella *larvae each were exposed to wasps until parasitization was observed. Individual larvae that had been attacked by the parasitoid were collected and fed on fresh cabbage leaves. Larval samples were taken at four different time intervals after parasitization (6, 12, 24 and 48 hrs post-parasitization) and the samples were kept at -80°C until RNA isolation. The same numbers of mixed larval instars (3^rd ^and 4^th^) of non-parasitized larvae were collected as the control treatment. It is worth mentioning that *P. xylostella *larvae parasitized at 3^rd ^instar continue to develop to 4^th ^instar.

### Sample preparation, deep sequencing and *de novo *transcriptome assembly

Total RNA was extracted from all larval samples using Tri-Reagent™ (Molecular Research Center Inc.). RNA extracted from larvae at various time points post-parasitization were pooled and therefore temporal expression data was lost. This was also performed for non-parasitized samples. The pooled RNA sample concentrations were measured using a spectrophotometer and integrity was ensured through analysis on a 1% (w/v) agarose gel. The samples with total concentration of 3.9 and 4.1 μg/μl for parasitized and non-parasitized larvae, respectively, were used for cDNA library production.

The cDNA library was prepared by using 5 μg of starting material for the Illumina mRNA Sequencing Sample preparation procedure (kit RS-930-1001). This involved purification and fragmentation of mRNA, first strand cDNA synthesis, second strand cDNA synthesis, end repair, addition of "A" bases to 3' ends, ligation of adapters, purification of ligated products, and PCR amplification to enrich cDNA templates. The library was validated, quantified and subjected to deep sequencing using a Genome Analyzer IIx Next generation sequencer on a 66 cycle single-end sequencing run, following the supplier's instructions (Geneworks, Adelaide). The GAII analyzer data were output as sequence tags of 65 bases. Sequence.txt files (in FASTQ format) were generated using Illumina Pipeline version 1.5.1. The CLC Genome Workbench (version 4.0.2) [75] algorithm for *de novo *sequence assembly was used to assemble contigs from a pooling of all the short-read data, using default parameters (similarity = 0.8, length fraction = 0.5, insertion/deletion cost = 3, mismatch cost = 3).

### RNA sequence analysis

The contigs arising from the *de novo *assembly were then used as a reference set of transcripts for RNAseq analysis. Short-read sequence data from parasitized and non-parasitized larvae were separately mapped against the reference set of assembled transcripts using the CLC Genome Workbench RNAseq function (min. length fraction = 0.9, maximum mismatches = 2). The relative transcript levels were output as RPKM (Reads Per Kilobase of exon model per Million mapped reads) values, which take into account the relative size of the transcripts and only uses the mapped-read datasets (i.e. excludes the non-mapped reads), to determine relative transcript abundance. In this way, the output for each dataset can be directly compared as the number of mapped reads per dataset and transcript size has already been taken into account.

Reads from parasitized and non-parasitized larvae were cleaned and combined, before *de novo *transcriptome assembly was carried out using Oases 0.1.18 [32]. The individual sets of reads were then mapped back to the transcripts using BWA 0.5.8a [76]. The average read depth (proportional to expression level) for each transcript was then calculated using SAMtools 0.1.8 [77]. The transcripts that had a greater than two-fold average read depth difference between the parasitized and non-parasitized sets were counted as being statistically biologically relevant and were selected for annotation. We used both CLC and Oases to compare assembly of contigs. In general, Oases produced similar contigs to CLC, although contig lengths produced by Oases were in some instances longer.

### BLAST homology search and annotation

BLASTX algorithm [78] with an E-value cut off of 10^-6 ^was applied to the National Centre for Biotechnology Information (NCBI) non-redundant protein sequence database, to determine the homology of sequences with known genes. In the absence of *P. xylostella *and *D. semiclausum *genome sequences, we discarded annotations that showed similarity to hymenopteran genes and tried to use annotations that showed the highest similarity to lepidopteran genes. Gene ontology and annotation were performed on all assembled contigs greater than 200 bp length by BLAST2GO software http://www.blast2go.org[79]. For gene ontology mapping, Blast2GO (which performs four different mapping strategies) was used, and the program defaults were applied for all annotation steps [79]. BLAST2GO allows the selection of a significance level for the False Discovery Rate (FDR), which was used as a cut-off at the 0.05% probability level. The data from InterPro terms [80], enzyme classification codes (EC), and metabolic pathways (KEGG, Kyoto Encyclopedia of Genes and Genomes) were merged with GO terms for a wide functional range cover in annotation.

For some of the identified *D. semiclausum *ichnovirus (*Ds*IV) genes, ORFs were predicted and identified by using ORF finder at NCBI http://www.ncbi.nlm.nih.gov/gorf/gorf.html. Predicted ORFs with highest BLASTp E-values in internal comparisons involving other IV genes, were accepted for further analyses.

### Quantitative RT-PCR (qRT-PCR) validation of deep sequencing data

Quantitative RT-PCR technique was used on the same RNA samples which were used for transcriptome profiling to verify deep sequencing results using three replicates, each obtained from a pool of 10 larvae. In addition, to observe gene expression levels at different time points after parasitization for a selected group of genes, another experiment was performed by parasitizing 2^nd ^instar *P. xylostella *larvae. For each time point after parasitization, a pool of 10 larvae was used. The RNA samples were extracted from larvae at 16, 24 and 48 hrs after parasitization.

First strand cDNA was synthesized from 1 μg of RNA using M-MuLV reverse transcriptase (New England BioLabs). The qPCR reaction consisted of 2 μL of diluted cDNA (10 ng), 5 μL of Platinum SYBR Green SuperMix-UDG with ROX (Invitrogen), and 1 μM of each primer (Table 5) in 10 μL total volume. Reactions were performed in triplicates in a Rotor-Gene thermal cycler (QIAGEN) under the following conditions: 50°C for 2 min; 95°C for 2 min; and 40 cycles of 95°C for 10 s, 60°C for 15 s, and 72°C for 20 s, followed by melting curve generation (68°C to 95°C). Melting curves for each sample were analyzed to check the specificity of amplification. Gene copy numbers were calculated using the Rotor-Gene software, and an endogenous actin reference gene was used for normalization.

**Table 5:** Primers used for qRT-PCR analyses to validate deep sequencing data

Gene	Forward primer	Reverse Primer
Storage protein 1	CAAGACACGCTACGACGC	GTCGGCATGACGAAGTAC
Insulin receptor	GTACCCCTCGATCTCGCG	CCCACGTCAAGGGAACCC
JH epoxide hydrolase	AGGATCTACGCGGAGGGC	TGGTACACCACTTCGTAC
Ecdysteroid regulated protein	AACCCGAAGAGCCGAAGC	CTCTGTAGTCGCTGCTAC
Cuticle protein	CAGGATGACGAGTCTGGC	GTCTGCCTCGTATTCTAC
Toll receptor	CCTCCGGCAACGCCCTAG	CGCACAGAAATTCAGAGG
Hemolin	AGCTCCAGAGACTACGCC	GTGTTGTAGGAACCATTG
Gloverin	AGCTAGCCCGGCATCCGC	GACGGTAGCCCGCCTTAC
pxSerpin 3	GAATAGCTTCTACTACGC	TGATAGCGAATTCGGTAC
Actin	ATGGAGAAGATCTGGCAC	GGAGCCTCCGTGAGCAGC

## Authors' contributions

KE performed experiments, analysed data and wrote the manuscript. RP, DS, LN analysed data and contributed in writing the manuscript. RVG reviewed the manuscript and provided insect resources. SA conceived the study, analysed data and wrote the manuscript. All authors read and approved the final manuscript.
